# Risedronate Attenuates Podocyte Injury in Phosphate Transporter-Overexpressing Rats

**DOI:** 10.1155/2019/4194853

**Published:** 2019-10-23

**Authors:** Yohei Asada, Takeshi Takayanagi, Tsukasa Kawakami, Eisuke Tomatsu, Atsushi Masuda, Yasumasa Yoshino, Sahoko Sekiguchi-Ueda, Megumi Shibata, Tomihiko Ide, Hajime Niimi, Eishin Yaoita, Yusuke Seino, Yoshihisa Sugimura, Atsushi Suzuki

**Affiliations:** ^1^Department of Endocrinology and Metabolism, Fujita Health University, Toyoake, Aichi 470-1192, Japan; ^2^Joint Research Support Promotion Facility, Center for Research Promotion and Support, Fujita Health University, Toyoake, Aichi 470-1192, Japan; ^3^Department of Anatomy, Fujita Health University, Toyoake, Aichi 470-1192, Japan; ^4^Department of Structural Pathology, Institute of Nephrology, Niigata University Graduate School of Medical and Dental Sciences, Niigata 950-2102, Japan

## Abstract

Osteoporosis patients with chronic kidney disease (CKD) are becoming common in our superaging society. Renal dysfunction causes phosphorus accumulation in the circulating plasma and leads to the development of CKD-mineral bone disorder (MBD). We have previously reported that type III Pi transporter-overexpressing transgenic (Pit-1 TG) rats manifest phosphate (Pi)-dependent podocyte injury. In the present study, we explored the effect of risedronate on Pi-induced podocyte injury *in vivo*. Pit-1 TG rats and wild-type rats at 5 weeks old were divided into a risedronate-treated group and an untreated group. We subcutaneously administered 5 *μ*g/kg body weight of risedronate or saline twice a week during the experimental period. Risedronate did not alter serum creatinine levels at 5, 8, and 12 weeks of age. However, electron microscopy images showed that thickening of the glomerular basement membrane was improved in the risedronate treatment group. Furthermore, immunostaining for podocyte injury markers revealed that both desmin- and connexin43-positive areas were smaller in the risedronate-treated group than in the untreated group, suggesting that bisphosphonates could rescue Pi-induced podocyte injury. In conclusion, our findings suggest that risedronate could maintain glomerular barrier function by rescuing Pi-induced podocyte injury.

## 1. Introduction

Phosphate (Pi) is known as an essential component for cell viability via ATP synthesis. On the other hand, Pi overload causes cell stress and induces apoptosis [1]. We have previously shown that high extracellular Pi induces cell death of vascular smooth muscle cells of rat A-10 [2]. Serum Pi level is mainly controlled by three major organs: kidney, bone, and intestine. Among them, reabsorption of Pi in the proximal tubules of the kidney is regulated by parathyroid hormone and other phosphaturic hormones such as fibroblast growth factor-23 [3]. In patients with chronic kidney disease (CKD), CKD-mineral and bone disorder (MBD) develops in association with secondary hyperparathyroidism owing to Pi accumulation and leads to an increased risk of fracture, cardiovascular events, and overall increased mortality [4–6]. Moreover, the Pi load itself contributes to the progression of renal insufficiency and vascular calcification [7–9]. Therefore, the regulation of serum Pi levels in hyperphosphatemia is considered important for improving life expectancy and preventing end-stage renal disease (ESRD).

Nephrotic syndrome, characterized by proteinuria, hypoalbuminemia, and dyslipidemia, occurs mainly because of failure of the glomerular filtration barrier mechanism [10]. The barrier mechanism is constructed of three essential components: the glomerular basement membrane (GBM), podocytes, and glomerular endothelial cells [11]. Among them, the terminally differentiated podocytes play a role as a critical size and charge barrier to prevent proteinuria. Podocyte injury brings on noticeable proteinuria with or without the loss of renal function [12]. Podocytes are injured by underlying diseases of nephrotic syndrome such as membranous nephropathy, minimal-change disease, and diabetic nephropathy [13–15]. We previously reported that transgenic (TG) rats overexpressing the type-III Na-dependent Pi transporter (Pit-1) develop spontaneous cataracts, bone loss, and nephrotic syndrome [16, 17]. In our Pit-1-overexpressing TG rats, their glomeruli showed thickened GBM and podocyte injury, suggesting that Pi overload could damage the glomerular barrier system through podocyte injury [16].

Nephrotic syndrome with persistent proteinuria results in serious complications and progressive kidney disease [10]. Glucocorticoid (GC) is effective in reducing proteinuria and halting the progress of kidney disease in several types of nephrotic syndrome. On the other hand, continuous GC treatment induces adverse side effects such as secondary diabetes and GC-induced osteoporosis (GIOP) [18, 19]. To prevent GIOP, bisphosphonates are currently the first-line drug [19, 20]. Bisphosphonates are very effective in reducing GIOP-associated bone fracture, but their use is restricted in patients with severe kidney dysfunction [21]. Animal experiments have shown that the use of risedronate is associated with increased risk of tubular degeneration and single-cell necrosis in the proximal convoluted tubules [22]. However, the effect of bisphosphonates on glomerulus disease such as nephrotic syndrome without severe kidney dysfunction has not yet been elucidated. In the present study, we examined the diachronic change in renal function in Pit-1 TG rats with risedronate administration to explore the effect of bisphosphonates on glomerular barrier function *in vivo*.

## 2. Materials and Methods

### 2.1. Animals

The mouse Pit-1 gene was ubiquitously expressed under the control of a cytomegalovirus early enhancer element and chicken *β*-actin (CAG) promoter in TG rats, and upon pronuclear DNA microinjection into rat zygotes [16]. Sodium risedronate hydrate was provided by EA Pharma Co. Ltd. (Tokyo, Japan). Male Pit-1 TG rats and their wild-type littermates (WT) were housed at 24°C with a 12 h/12 h light/dark cycle and were allowed free access to normal rodent chow and water. They were randomly divided into the following four groups: WT rats with risedronate administration (WT-R, *n* = 13), WT rats without risedronate administration (WT-C, *n* = 14), Pit-1 TG rats with risedronate administration (TG-R, *n* = 14), and Pit-1 TG rats without risedronate administration (TG-C, *n* = 15). Risedronate (5 *μ*g/kg body weight) was subcutaneously administered twice a week from 5 weeks old through 12 weeks old. The dose of risedronate chosen was previously determined as an effective pharmacological dose used in animal experiments by Matheny et al. [23]. The equivalent volume of saline was administered to the control groups without risedronate administration. Blood samples were taken from the tail vein. These samplings were conducted at 5, 8, and 12 weeks of age. Routine serum chemistries were measured using a Hitachi 7180 automatic analyzer (Hitachi High Technologies, Tokyo, Japan). Tissues for histological analysis by light microscopy and electron microscopy were collected at 8 and 12 weeks of age. All animal procedures and a statement of protocol were approved by the Institutional Animal Care and Use committee and government authorities.

### 2.2. PCR

Genotyping of the transgenic animals was carried out by PCR. Genomic DNA purified from tail clips was digested with proteinase K and hybridized with a specific probe for mouse Pit-1. For subsequent genotyping by PCR, we used the forward primer 5′-ACGGCTTGATAGATGTGG-3' (681–698 of M73696) and the reverse primer 5′-TAAGGGACTTTCAGACGG-3' (1234–1217 of M73696). For genotyping, PCR was performed at 95°C for 30 s, 60°C for 30 s (30 cycles), and 72°C for 1 min.

### 2.3. Light Microscopy

Kidney samples were fixed in 10% neutral-buffered formalin, embedded in paraffin, and cut into sections of 2-*μ*m to 3-*μ*m thickness. The sections were stained with hematoxylin and eosin or periodic acid-Schiff (PAS). Using light microscopy at low power magnification, a plan of suitable sections was drawn with the position of every glomerulus marked, which generally covered the whole section. At high power magnification, glomeruli were examined for lesions.

### 2.4. Electron Microscopy

Small pieces of tissue, 2 × 4 mm, were fixed in 2.5% glutaraldehyde (GA)/0.05 M sodium phosphate, at pH 7.4 (PB), for 2 h. After washing with PB, samples were dehydrated in a graded ethanol series and treated with *n*-butyl glycidyl ether before being embedded in Epon 812 (Taab Laboratories, Aldermaston, UK). Ultrathin sections (0.1 *μ*m) were doubly stained with 2% uranyl acetate and 1% lead citrate and then observed using a JEM-1010 (JEOL, Tokyo, Japan) transmission electron microscope (TEM) at an accelerating voltage of 80 kV. The TEM images were analyzed using image-J, and the width of the GBM was measured between the cytoplasmic membranes right under the foot processes and the glomerular endothelial cells. The intervals of measurement were 1 micrometer along the longitudinal axis of glomerular basement membranes.

### 2.5. Immunofluorescence Microscopy

The indirect immunofluorescence technique was applied to frozen kidney sections and outgrowths from glomeruli as described previously [24]. In brief, the rat kidneys were snap-frozen at −70°C, sectioned at a thickness of 3 *μ*m in a cryostat, and fixed in 2% paraformaldehyde in phosphate-buffered saline (PBS) for 5 min. The primary antibodies used were as follows: monoclonal anti-nephrin antibody (5-1-6; courtesy of Dr. H. Kawachi, Niigata University, Niigta, Japan) [25]; murine monoclonal anti-desmin antibody (clone D-33; Dako Cytomation, Glostrup, Denmark); rabbit anti-connexin43 antibody (Sigma-Aldrich, St Louis, MO, USA); murine monoclonal anti-ZO-1 antibody (Zymed Laboratories, South San Francisco, CA, USA); and rabbit anti-laminin antibody (Dako Cytomation). For double-label immunofluorescence microscopy, monoclonal anti-nephrin antibody and rabbit anti-laminin antibody, murine monoclonal anti-desmin antibody and rabbit anti-laminin antibody, and rabbit anti-connexin43 antibody and murine monoclonal anti-ZO-1 antibody were mixed and applied simultaneously as primary antibodies. After washing with PBS, the sections were stained with fluorescein isothiocyanate (FITC)- or tetramethyl rhodamine isothiocyanate (TRITC)-conjugated anti-rabbit IgG, rewashed with PBS and subsequently reacted with FITC- or Texas Red-conjugated anti-mouse IgG. PBS, normal rabbit serum, or murine IgG monoclonal antibody (against rotavirus), shown not to react with rat glomeruli, were used as negative controls for the primary antibodies. 4′,6-diamidino-2-phenylindole and dihydrochloride (Dojindo, Kumamoto, Japan) were used for nuclear staining. Immunofluorescence of the sections was observed using a laser-scanning confocal microscope (A1Rsi; Nikon, Tokyo, Japan).

### 2.6. Statistical Analysis

Results are expressed as means ± SD. Data were analyzed using JMP ver.13.0. Statistical analyses were performed by ANOVA and Tukey HSD tests. The level of significance was set at *P* < 0.05.

## 3. Results

Risedronate did not affect serum creatinine levels of both WT and TG rats (Table 1). Serum calcium levels in the TG-R group were lower than in the WT-R group at 5 weeks, but reached the same level as other groups at 8 and 12 weeks of age. Serum phosphorus and creatinine levels among all four groups were identical.

**Figure 1:**
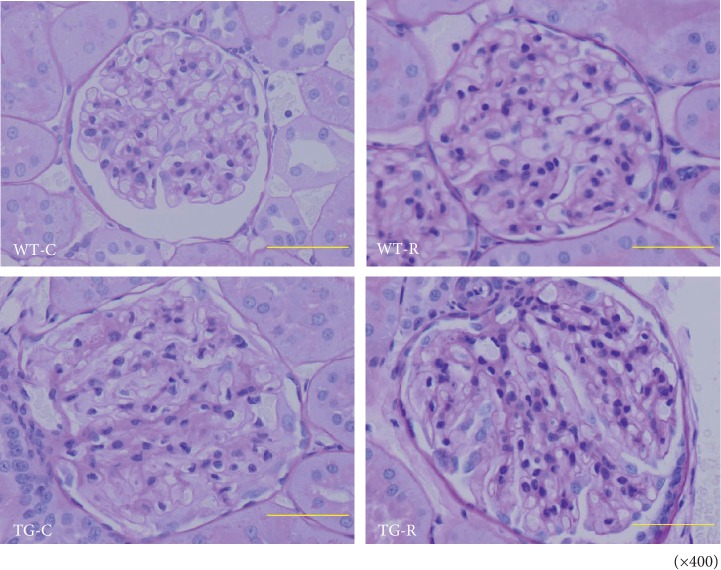
Light microscopy following periodic acid-Schiff staining in the kidney from WT-C, WT-R, TG-C, and TG-R rats at 12 weeks of age. Experimental groups were as follows: wild-type littermates (WT) without risedronate administration (WT-C), WT rats with risedronate administration (WT-R), Pit-1 TG rats with risedronate administration (TG-R), and Pit-1 TG rats without risedronate administration (TG-C). Original magnification: ×400. Bar 50 *μ*m.

**Figure 2:**
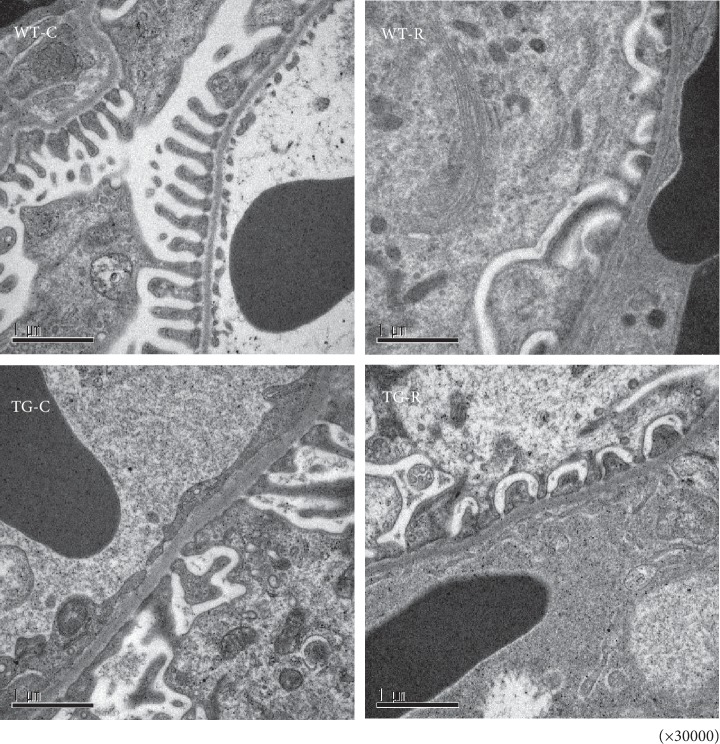
Ultrastructural analysis of glomeruli from WT-C, WT-R, TG-C, and TG-R rats by transmission electron microscopy at 12 weeks of age. Experimental groups were as follows: wild-type littermates (WT) without risedronate administration (WT-C), WT rats with risedronate administration (WT-R), Pit-1 TG rats with risedronate administration (TG-R), and Pit-1 TG rats without risedronate administration (TG-C). Original magnification: ×30,000.

**Figure 3:**
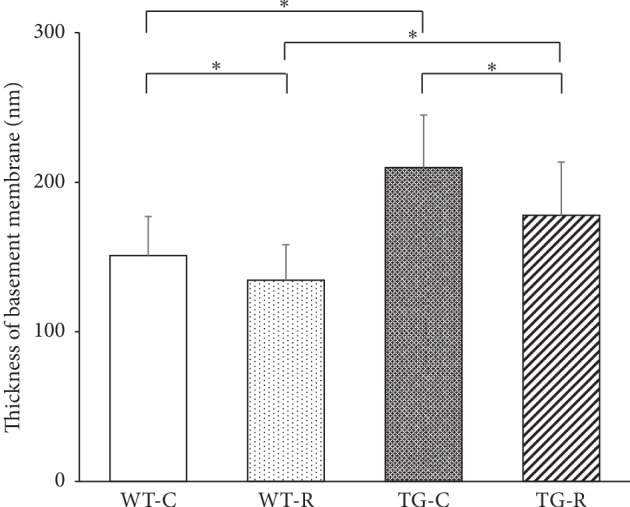
Thickness of the glomerular basement membrane measured by ultrastructural analysis under transmission electron microscopy. Experimental groups were as follows: wild-type littermates (WT) without risedronate administration (WT-C), WT rats with risedronate administration (WT-R), Pit-1 TG rats with risedronate administration (TG-R), and Pit-1 TG rats without risedronate administration (TG-C). ^*∗*^*P* < 0.0001.

Histological examination of kidney sections with PAS staining at 8 and 12 weeks of age showed hyaline and vacuolar degeneration which were conspicuously observed in both TG groups but not in control groups (Figure 1). Hyaline droplet-positive lesions were observed in almost half of glomeruli. Tubular atrophy and interstitial cell infiltration were infrequent. Ultrastructural analysis by TEM showed podocyte effacement and thickness of GBM in the kidney of 12-week-old TG-C rats (Figure 2). In addition, TG-C rats had lost the three-layered structure of the GBM at 12 weeks of age. When risedronate was administered to TG rats (TG-R), podocyte effacement was rescued, and the GBM in TG-R rats retained its three-layered structure (Figure 2). The width of the GBM in TG-R rats was significantly narrower than that in TG-C rats (Figure 3).

**Figure 4:**
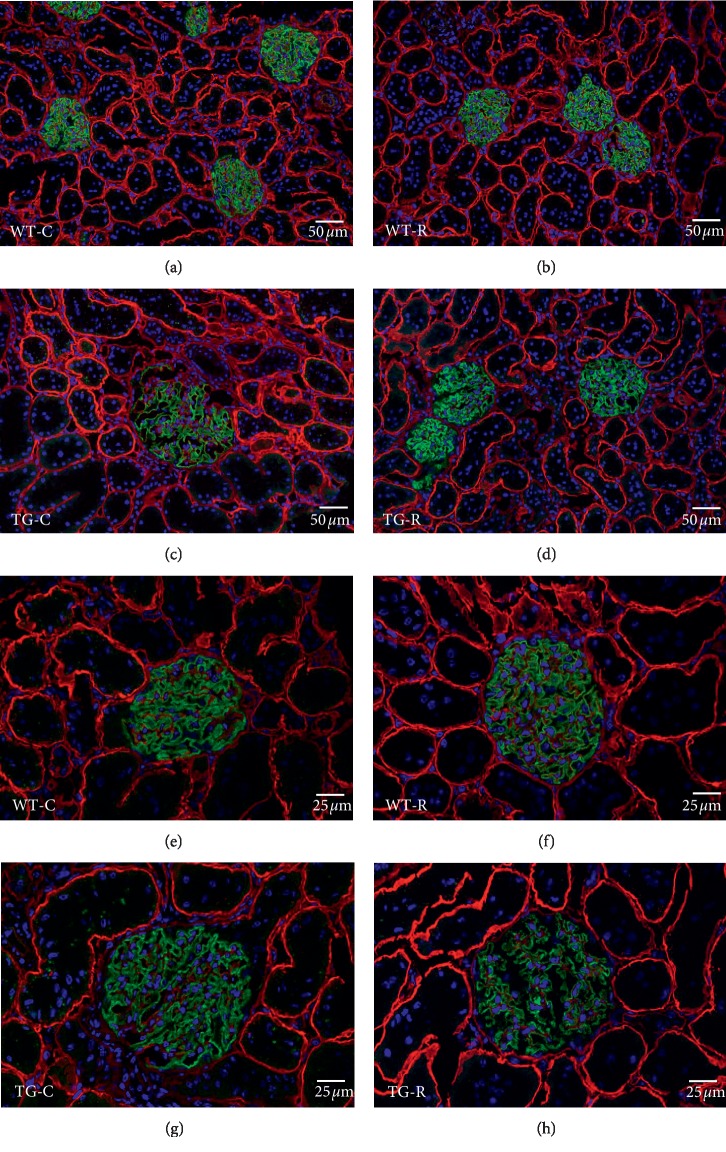
Immunofluorescence microscopy for nephrin (green) in glomeruli of WT-C (a and e), WT-R (b and f), TG-C (c and g), and TG-R rats (d and h) at 12 weeks of age. Double-labeled immunostaining for laminin (red) was carried out to locate the glomerular capillary wall. Experimental groups were as follows: wild-type littermates (WT) without risedronate administration (WT-C), WT rats with risedronate administration (WT-R), Pit-1 TG rats with risedronate administration (TG-R), and Pit-1 TG rats without risedronate administration (TG-C). Bar = 50 *μ*m (a, b, c, and d). Bar = 25 *μ*m (e, f, g, and h).

**Figure 5:**
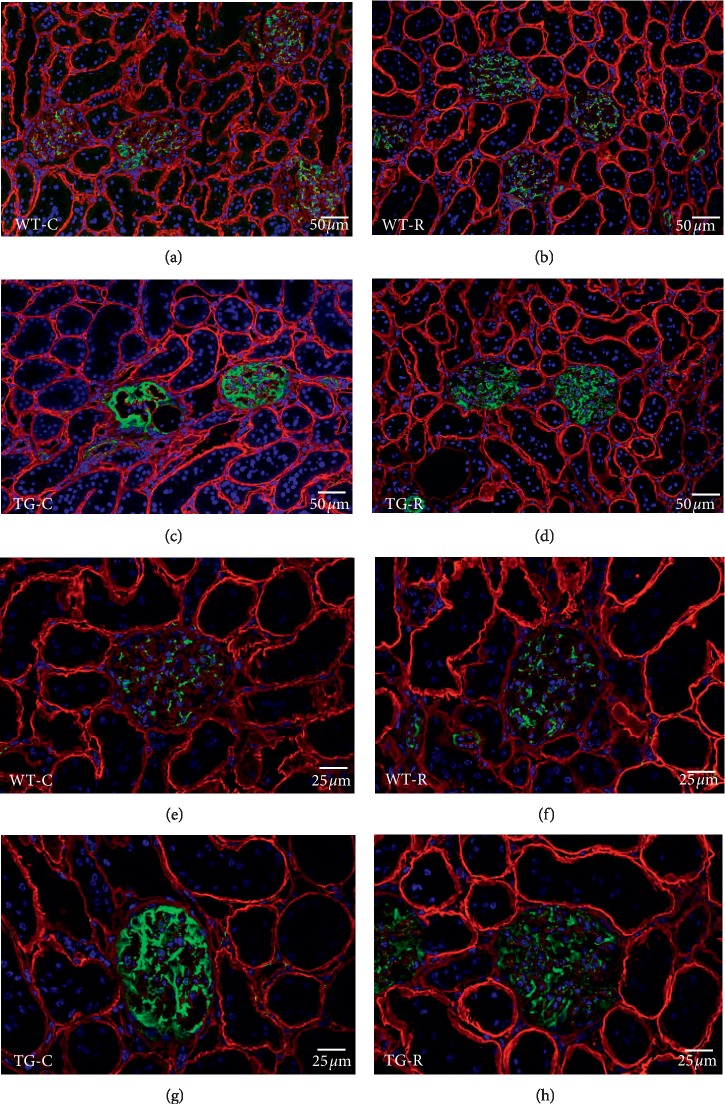
Immunofluorescence microscopy for desmin (green) in glomeruli of WT-C (a and e), WT-R (b and f), TG-C (c and g), and TG-R rats (d and h) at 12 weeks of age. Double-labeled immunostaining for laminin (red) was carried out to locate the glomerular capillary wall. Experimental groups were as follows: wild-type littermates (WT) without risedronate administration (WT-C), WT rats with risedronate administration (WT-R), Pit-1 TG rats with risedronate administration (TG-R), and Pit-1 TG rats without risedronate administration (TG-C). Bar = 50 *μ*m (a, b, c, and d). Bar = 25 *μ*m (e, f, g, and h).

**Figure 6:**
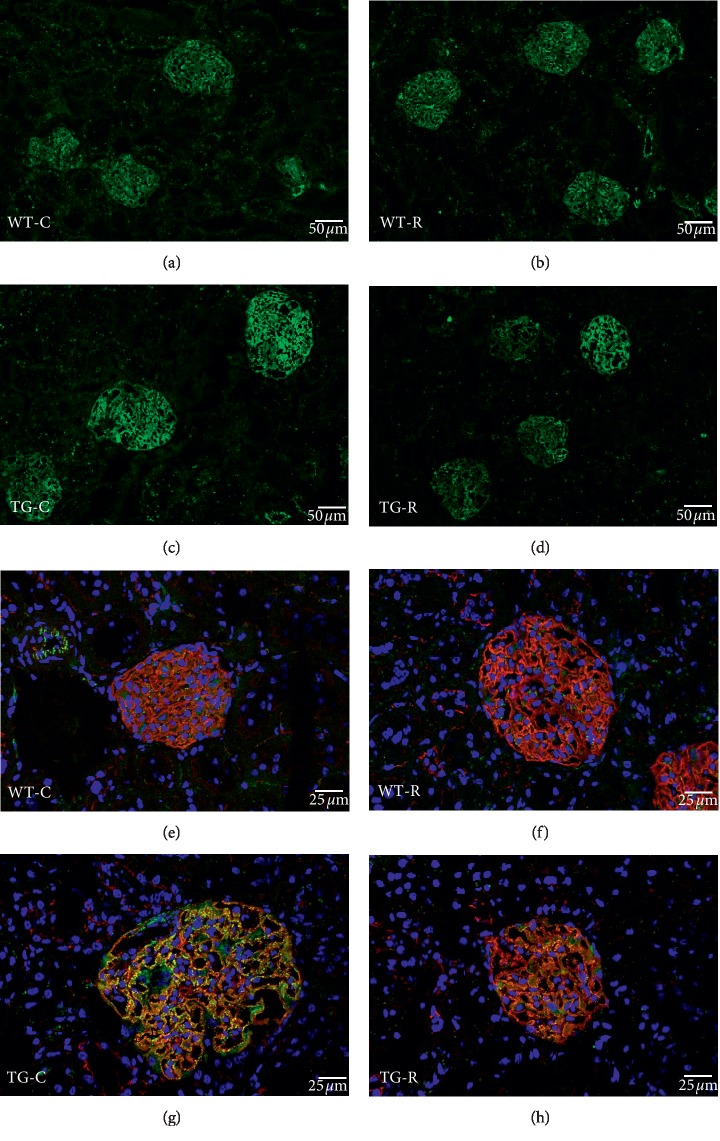
Immunofluorescence microscopy for connexin43 (green) in glomeruli of WT-C (a and e), WT-R (b and f), TG-C (c and g), and TG-R rats (d and h) at 12 weeks of age. Double-labeled immunostaining for tight junction protein ZO-1 (red in E-H) was carried out to locate the glomerular capillary wall. Experimental groups were as follows: wild-type littermates (WT) without risedronate administration (WT-C), WT rats with risedronate administration (WT-R), Pit-1 TG rats with risedronate administration (TG-R), and Pit-1 TG rats without risedronate administration (TG-C). Bar = 50 *μ*m (a, b, c, and d). Bar = 25 *μ*m (e, f, g, and h).

By immunofluorescence microscopy, we examined podocyte constituent proteins (nephrin) and podocyte injury markers (desmin and connexin43) at the age of 12 weeks. The localization and staining intensity of nephrin were the same among all four groups (Figure 4). In contrast, immunostaining for podocyte injury markers showed remarkable differences between the TG groups. The TG-C group showed wider and clearer desmin-enhanced areas than the WT groups. In the TG-R group, the area was obviously narrower and weaker than in the TG-C group (Figure 5). Connexin43-enhanced areas were observed as a dotted pattern along the glomerular capillary wall in the TG-C group, while in the TG-R group, the area exhibited fewer dots with connexin43 staining than the TG-C group (Figure 6).

## 4. Discussion

In the present study, we found that risedronate administration could save the barrier mechanisms of the glomerulus in Pit-1 TG rats. Although bisphosphonates, including risedronate, are contraindicated for severe renal insufficiency, our study showed that they exert a protective effect on glomeruli in the kidney affected by Pi overload.

Nephrotic syndrome is characterized by massive proteinuria with damaged barrier function of the glomerulus [26]. Pathological proteinuria is associated with both effacement of foot processes and the loss of slit diaphragms [27, 28]. Podocytes cover the GBM, and their interdigitated foot processes interlink with slit diaphragms. Both the GBM and the slit diaphragms are essential components of the glomerular filtration barrier, which prevents protein loss into the urine. The GBM is the central, noncellular layer of the glomerular filtration barrier that is situated between the two cellular components: the fenestrated endothelial cells and the interdigitated podocyte foot processes. The GBM is synthesized and repaired by podocytes postnatally, and GBM abnormality could indicate podocyte dysfunction [29, 30]. We have shown that risedronate administration improves thickening of the GBM and maintains its three-layered structure in Pit-1 TG rats. In addition, risedronate attenuates the expression of podocyte injury markers such as desmin and connexin43 in Pit-1 TG rats. These results suggest that risedronate rescues glomerular barrier function by protecting against Pi-induced podocyte injury. Bisphosphonates including risedronate are currently the most important class of antiresorptive agents used in the treatment of metabolic bone diseases including primary and secondary osteoporosis, tumor-associated osteolysis, and hypercalcemia [31]. Nephrotic syndrome is often treated with GC, and GIOP and subsequent fractures are a significant concern in these patients [19, 20]. Bisphosphonates are effective in treating GIOP and are considered as the first-line drug to prevent fracture due to GIOP [19, 20]. Theoretically, bisphosphonates should be administered to nephrotic syndrome patients with GIOP, but all bisphosphonates carry warning labels or contraindications against use in patients with severe renal dysfunction [21]. A histopathological study revealed that the proximal convoluted tubules are the primary target for renal injury caused by bisphosphonates, and tubular degeneration and single-cell necrosis of these tubules were observed with bisphosphonate administration in a rat experimental model [22]. However, the effect of bisphosphonates on the glomerulus and its barrier function has not yet been elucidated. In this study, we show that the serum creatinine level was not elevated with risedronate administration. Moreover, no renal tubule necrosis was observed. In fact, risedronate improved Pi-induced damage to the barrier mechanism in Pit-1 TG rats. These results suggest that risedronate could be safe and show a rather beneficial effect on nephrotic syndrome without ESRD.

Inorganic phosphate (Pi) is a key element of skeletal mineralization and an essential nutrient for the maintenance of cell viability through the process of energy metabolism, producing ATP [32]. The ubiquitous expression of Pit-1 is compatible with housekeeping maintenance of intracellular Pi homeostasis by transporting Pi from interstitial fluid for normal cellular functions [33]. Pit-1 knockout mice, generated by homologous recombination in embryonic stem cells, died around midgestation [34]. Thus, Pit-1 seems to play an essential role in cell survival and tissue development. In contrast, accumulating evidence suggests that continuous Pi overload of nonskeletal tissues from the extracellular milieu causes cell stress [1]. In the clinical setting, high plasma Pi is an independent risk factor for an accelerated decline in renal function and higher mortality in predialysis patients [8]. Maintenance of Pi within the normal range is one of the crucial factors in preventing renal failure in CKD patients. Nowadays, CKD-MBD is a critical concept in achieving better prognosis in the superaging population because renal function decreases with aging. In the aging population, comorbidities with CKD and osteoporosis are more common than in the younger generation [35], and the number of bisphosphonate users with CKD is increasing. Therefore, our results showing a beneficial effect of bisphosphonate on glomerular barrier function might contribute to better prognosis of CKD-MBD patients without ESRD. Thus, the use of bisphosphonates in osteoporosis patients with moderate CKD may not only prevent osteoporotic fractures but also protect glomerular function rather than impair renal function.

There are three possible renoprotective mechanisms of bisphosphonate in this study:

### 4.1. Inhibition of Direct Pi Uptake by Glomerular Epithelial Cells

Bisphosphonates are in a nonpolar lipophilic configuration under the acidic environment of the resorption pit beneath the ruffled borders of osteoclasts, and they transverse the cytoplasmic membrane of the osteoclast specifically [36]. In a neutral environment, they assume a polar hydrophilic configuration, and they use transporters to cross the cytoplasmic membrane of nonosteoclastic cells. Bisphosphonates are considered to enter into soft-tissue cells via SLC20 and/or SLC34 Pi transporters [37]. Therefore, bisphosphonates could compete with Pi by passing from the extracellular fluid into the cytoplasm through Pit-1 and rescue podocytes from Pi stress.

### 4.2. Suppression of Bone Resorption by Bisphosphonate Could Reduce the Outflow of Pi into the Blood, Resulting in Relief of the Pi Burden on the Kidney

Ibandronate treatment did not significantly affect the increase in serum levels of Pi, creatinine, blood urea nitrogen, or PTH in uremic rats. However, their artery calcification was prevented, and this correlated with decreasing serum bone Gla protein (BGP; osteocalcin), a marker of an increased bone turnover [38]. Therefore, unchanged Pi concentration in the present study cannot preclude an indirect effect of bisphosphonate on podocytes.

### 4.3. Inhibition of Activation of the Rho-Rho Kinase-Dependent Pathway by Inhibition of Farnesyl Pyrophosphate Synthase (FPPS)

FPPS is known to be expressed in kidney cells, where it activates small GTPases including Rho kinase [39]. Rho kinase is an effector molecule of Rho A, and Rho A plays an important role in maintaining the integrity of the glomerular filtration barrier under basal conditions. However, enhancement of Rho A activity above basal levels promotes podocyte injury [40]. Activation of Rho A in podocytes leads to albuminuria accompanied by a range of histologic changes characteristic of minimal-change disease and focal segmental glomerulosclerosis in humans. Although most changes are reversible, severe and prolonged activation of Rho A may cause irreversible glomerulosclerosis [41]. Accumulating evidence shows that activation of Rho kinase impairs podocyte function. Podocyte injury in the progression of diabetic nephropathy could also be mediated by activation of Rho kinase [42]. Nitrogen-containing bisphosphonates including risedronate could inhibit FPPS, thus leading to inhibition of Rho kinase. Therefore, bisphosphonates might exert a cytoprotective effect on podocytes through inhibition of the Rho-Rho kinase pathway in podocytes.

We have previously shown that Pi uptake of primary cultured podocytes from Pit-1 TG rats is higher than WT rats [16], and we found that urinary Pi excretion was elevated in Pit-1 TG rats regardless of risedronate treatment (data not shown). Therefore, higher urinary Pi excretion in TG rats could be burden on podocytes in Pit-1 TG rats, and it is likely that risedronate protects podocytes from Pi stress by not systemic but local mechanisms. To explore precise mechanisms of risedronate on Pi-induced podocyte injury, further examination such as by molecular biology technique would be required.

## 5. Conclusion

Our findings suggest that risedronate can maintain the glomerular barrier function by rescuing phosphate-induced podocyte injury.

## Figures and Tables

**Table 1:** Serum creatinine, calcium, and phosphate levels in study groups.

	Age (weeks)	WT-C (*n* = 14)	WT-R (*n* = 13)	TG-C (*n* = 15)	TG-R (*n* = 14)
Serum creatinine (mg/dL)	5	0.25 ± 0.03	0.24 ± 0.02	0.26 ± 0.06	0.23 ± 0.04
	8	0.36 ± 0.05	0.34 ± 0.06	0.33 ± 0.08	0.30 ± 0.06
	12	0.47 ± 0.07	0.44 ± 0.03	0.42 ± 0.07	0.42 ± 0.06

Serum calcium (mg/dL)	5	10.23 ± 0.65	10.22 ± 0.52	9.69 ± 0.77	9.53 ± 0.62^*∗*^
	8	10.66 ± 0.52	10.47 ± 0.36	10.31 ± 0.77	10.26 ± 0.54
	12	10.79 ± 0.42	10.58 ± 0.67	10.85 ± 1.01	10.98 ± 0.93

Serum phosphate (mg/dL)	5	9.33 ± 0.95	9.66 ± 0.99	10.53 ± 2.23	9.03 ± 1.13
	8	9.41 ± 1.57	8.78 ± 0.87	10.43 ± 2.97	9.50 ± 1.67
	12	9.43 ± 1.74	9.05 ± 2.23	11.85 ± 5.12	12.14 ± 4.10

Rats were divided into experimental groups as follows: wild-type littermates (WT) without risedronate administration (WT-C), WT rats with risedronate administration (WT-R), type III Pi transporter-overexpressing transgenic (Pit-1 TG) rats with risedronate administration (TG-R), and Pit-1TG rats without risedronate administration (TG-C). Data are presented as mean ± SD. ^*∗*^*P* < 0.05 compared with WT-R group.

## Data Availability

The data used to support the findings of this study are available from the corresponding author upon request.
